# Phylogeographic investigation of 2014 porcine epidemic diarrhea virus (PEDV) transmission in Taiwan

**DOI:** 10.1371/journal.pone.0213153

**Published:** 2019-03-06

**Authors:** Ming-Hua Sung, Chao-Nan Lin, Ming-Tang Chiou, I-Ju Cheng, Quang-Hien Thanh, Day-Yu Chao, Yu-Ching Lan

**Affiliations:** 1 Graduate Institute of Microbiology and Public Health, National Chung Hsing University, Taichung, Taiwan; 2 Department of Veterinary Medicine, National Pingtung University of Science and Technology, Pingtung, Taiwan; 3 Department of Health Risk Management, China Medical University, Taichung, Taiwan; 4 Graduate Institute, International Public Health Program, China Medical University, Taichung, Taiwan; 5 Big Data and Cyber Security Division, Walsin Lihwa Corporation, Taipei, Taiwan; Sun Yat-Sen University, CHINA

## Abstract

The porcine epidemic diarrhea virus (PEDV) that emerged and spread throughout Taiwan in 2014 triggered significant concern in the country’s swine industry. Acknowledging the absence of a thorough investigation at the geographic level, we used 2014 outbreak sequence information from the Taiwan government’s open access databases plus GenBank records to analyze PEDV dissemination among Taiwanese pig farms. Genetic sequences, locations, and dates of identified PEDV-positive cases were used to assess spatial, temporal, clustering, GIS, and phylogeographic factors affecting PEDV dissemination. Our conclusion is that S gene sequences from 2014 PEDV-positive clinical samples collected in Taiwan were part of the same Genogroup 2 identified in the US in 2013. According to phylogenetic and phylogeographic data, viral strains collected in different areas were generally independent of each other, with certain clusters identified across different communities. Data from GIS and multiple potential infection factors were used to pinpoint cluster dissemination in areas with large numbers of swine farms in southern Taiwan. The data indicate that the 2014 Taiwan PEDV epidemic resulted from the spread of multiple strains, with strong correlations identified with pig farm numbers and sizes (measured as animal concentrations), feed mill numbers, and the number of slaughterhouses in a specifically defined geographic area.

## Introduction

Porcine epidemic diarrhea virus (PEDV) causes acute diarrhea, vomiting, and dehydration, resulting in high mortality rates for suckling piglets [1]. Since 2010, a new PEDV variant belonging to Genogroup 2 has spread throughout the United States and across multiple Asian countries, including China and Taiwan [2, 3]. An initial identification in the US was made in April of 2013 [4]. A 40.5% premises-level incidence of PEDV caused the deaths of more than 8 million newborn piglets in a single year—an event that significantly affected the American swine industry [5–9]. Subsequent Genogroup 2 epidemics have been reported in major swine-producing countries such as Canada, Mexico, Taiwan, Korea and Japan [8, 10–13]. One of the most notable PEDV outbreaks occurred in South Korea in late 2013 [14], more than a half million pigs died from PEDV infections in Japan between 2013 and 2016 [15], and a significant increase in PEDV outbreaks occurred in Taiwan around the same time [16]. PEDV is now considered the world’s most catastrophic swine disease, with major financial impacts noted throughout the global pork industry.

The PEDV genome is comprised of at least seven open reading frames (ORF1a, ORF1b and ORF2-6) encoding four structural (S), envelope (E), membrane (M), and nucleocapsid (N) proteins [17]. A high degree of genetic diversity has been observed in the S glycoprotein gene [18–20]. Partial spike (S) polyprotein genes located in the virus envelope are central to PEDV biological properties such as interactions with cellular receptors during virus entry, the neutralizing of antibody induction in natural hosts, growth adaptation in vitro, and virulence attenuation in vivo [19]. The PEDV spike (S) protein is a type 1 transmembrane envelope glycoprotein with a 4,158 nucleotide sequence divided into S1 and S2 domains. The S1 region (aa 26–734) is responsible for viral binding, while the S2 domain (aa 735–1383) serves as an anchor for viral membrane and fusion activity [21, 22]. Thus, the S glycoprotein is considered a primary target for PEDV vaccine development. As the major envelope glycoprotein found in virion, S serves as an important viral component for studying genetic relationships among PEDV isolates, and for determining PEDV epidemiological status [23, 24].

PEDV is believed to infect pigs by both direct and indirect fecal-oral routes. Due to the scales and complexities of modern swine production systems, PEDV is likely transmitted between farms via diarrheic feces or vomitus; contaminated environmental sources involving clinically or sub-clinically infected pigs; trailers used to transport livestock, manure, or food sources; farmers or visitors wearing contaminated clothes; or wild animals and birds [5, 25]. Other potential sources include contaminated fomites (e.g., raw food, feed, sow milk), food ingredients or additives, and environmental features such as wind direction, farm altitude, terrain slope, and tree coverage [26]. After an initial outbreak, PEDV may spread at an increasingly rapid rate due to inadequate farm hygiene management procedures such as improper disinfection and poor biosecurity. The virus can remain dormant in weaning pigs or growth finishing units, eventually triggering mild symptoms and resulting in low mortality rates [6].

Although researchers believe that PEDV infections primarily result via fecal-oral routes, the rapid regional spread of the disease raises the possibility of airborne transmission [4]. Support for this hypothesis includes an identified correlation between disease-spread direction and prevailing wind direction [7], with environmental features such as land coverage, altitude, and slope possibly influencing airborne disease dissemination [26]. To determine specific temporal and geographic relationships associated with PEDV strain transmission, we used phylogenetic, phylodynamic and phylogeographic methods to systematically evaluate potential temporal and spatial transmission routes among Taiwanese swine farms during the 2014 outbreak.

## Materials and methods

### Sample collection

Epidemiological and geographic data were collected from 92 animal lab reports of PEDV viral infections involving 8,557 pig farms, 37 pig feed mills, 57 slaughterhouses, and 5,806,237 animals. These reports are available from an open database maintained by the Taiwan government. Additional epidemiological and genetic information was gathered for purposes of determining details of the disease spread. A total of 48 global PEDV whole genome sequences (S1 Table) and 49 Taiwan partial S1 gene sequences (**648 nucleotide of PEDV S1 gene position 1468–2115**) (S2 Table) were downloaded from GenBank, and information for the TW4 whole genome sequence was collected from a previous study [27]. Information datasets focused on pig feeding and disease were collected from the Taiwan open data website (http://data.gov.tw/), the Animal Health Research Institute of the Taiwan Executive Yuan’s Agricultural Council (http://eng.nvri.gov.tw/fmodule/Default.aspx), and GenBank (https://www.ncbi.nlm.nih.gov/genbank/).

In the first stage of this study, PEDV phylogenetic and phylogeographic data analyses were performed for purposes of organizing viral transmission evidence and tracking possible transmission routes. In the second, data for variables of interest associated with the pig feed industry were collected and combined with geographic information system (GIS) data for investigation using open source Quantum GIS (QGIS v3.2.3) software [28]. An SAS MIXED procedure was used to create linear regression models, with livestock breeding variables employed to predict PEDV infections.

### Phylogenetic data analysis

Gene sequences for the 49 global PEDV whole genomes and 49 Taiwan PEDV partial S1 gene sequences were downloaded from GenBank and aligned with a single Taiwan PEDV complete genome from a previous study [27] using the ClustalW multiple alignment feature in BioEdit [29]. The best-fit model, as determined using jModelTest 2.1.7 [30], had a gamma distribution (GTR+G+I). To ensure topological consistency, phylogenetic trees were constructed using maximum likelihood (ML) and Bayesian methods (MEGA version 6.0 and BEAST v1.8.2, respectively). Branch support was evaluated using bootstrap analyses based on 1,000 ML tree replications. Bootstrap values >75% were considered as belonging to the same monophyletic group.

### Phylodynamic and phylogeographic analyses

To identify the specific locations of migration events, we grouped the 49 PEDV isolates into different counties and used various PEDV viral strains as references. Spatial location reconstruction and viral migration activity were estimated using discrete coalescent tree and Bayesian phylogeographic methods. Bayesian Markov Chain Monte Carlo (MCMC) sampling using BEAST v1.8.2 [31] was employed to infer the time-scaled phylogenies of partial PEDV S genes. HKY+G and relaxed clock exponential models were used prior to setting coalescent population constants in the MCMC simulations. Estimated convergence and effective sampling sizes were visually assessed using Tracer v1.6. Multiple chains were combined based on a 10% burn-in using the version of LogCombiner (v1.8.2) included in the BEAST package. Maximum clade credibility trees with temporal and spatial annotations were summarized with the 10% burn-in removed using TreeAnnotator (v1.8.2, also in the BEAST package). FigTree (v1.4.2) was used to generate presentation figures.

Bayes factor (BF) tests were conducted to build statistical support for transmission routes among geographic locations using SPREAD3 (v0.9.6; BF cutoff = 3) [32]. BF values were used to indicate differences between posterior and prior probabilities so that rates between any two locations were non-zero. Routes with high BF values were considered as having greater potential for viral strain migration.

### Visualizing phylogeographic diffusion

To create animations of viral dispersion over time, annotated MCC trees were converted into a keyhole markup language (KML) file using SPREAD3 (v0.9.6). The KML file can be visualized with an open-access Earth map downloaded from Natural Earth (http://www.naturalearthdata.com) as a QGIS software base layer.

## Results

### PEDV genotype

**Fig 1:**
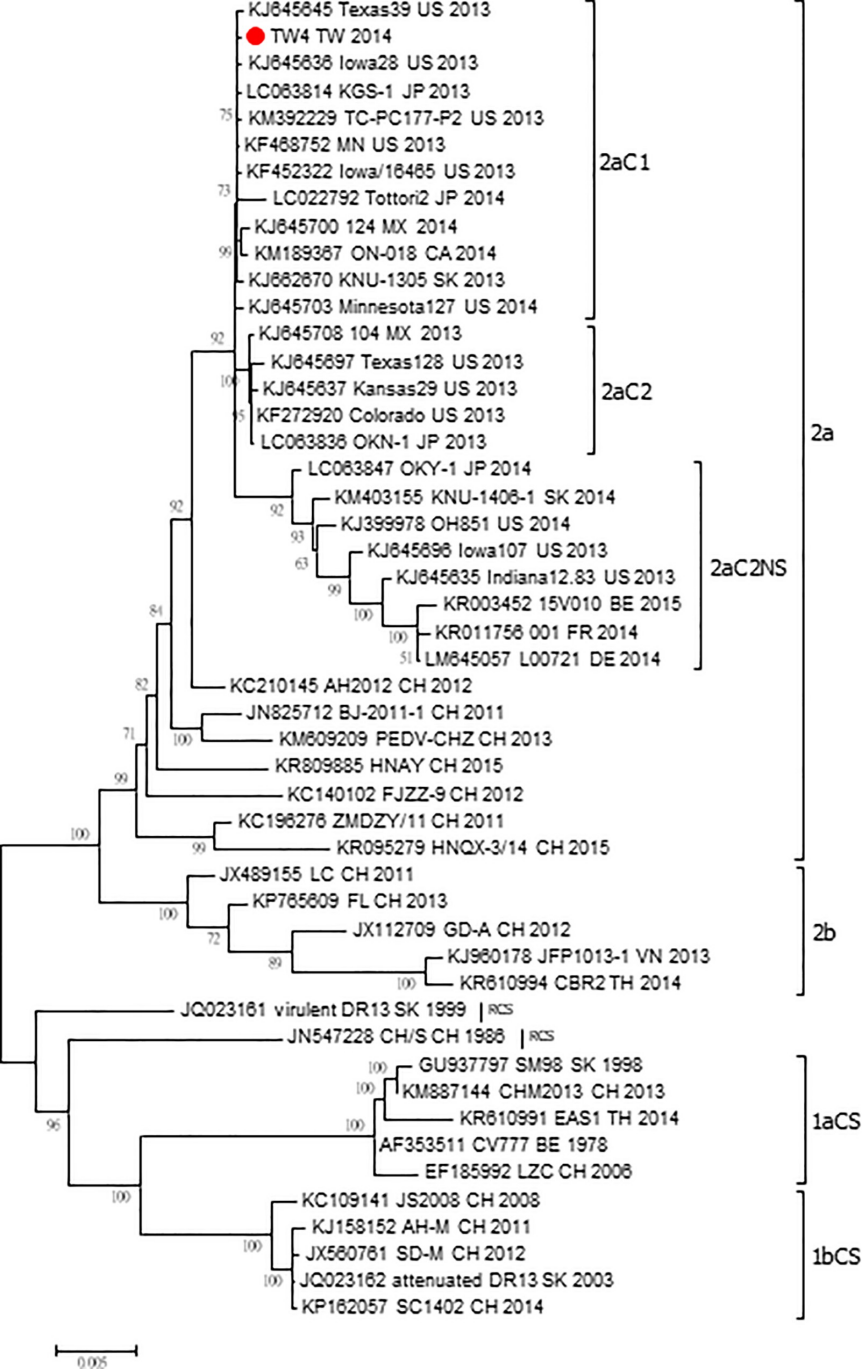
Data for PEDV49 whole genome phylogenetic relationships from ML tree GTR+G+I. A phylogenetic analysis was performed using data for 49 whole genome sequences from the countries listed in S1 Table. Maximum likelihood and Bayesian methods (MEGA 6.0 software) were used for tree construction. An interior branch test (1,000 replicates) was performed to determine internal node reliability. Scale bar indicates nucleotide substitutions per site. Monophyletic groups were identified as having bootstrap values >75%. Strains shown in box were collected in Taiwan.

**Fig 2:**
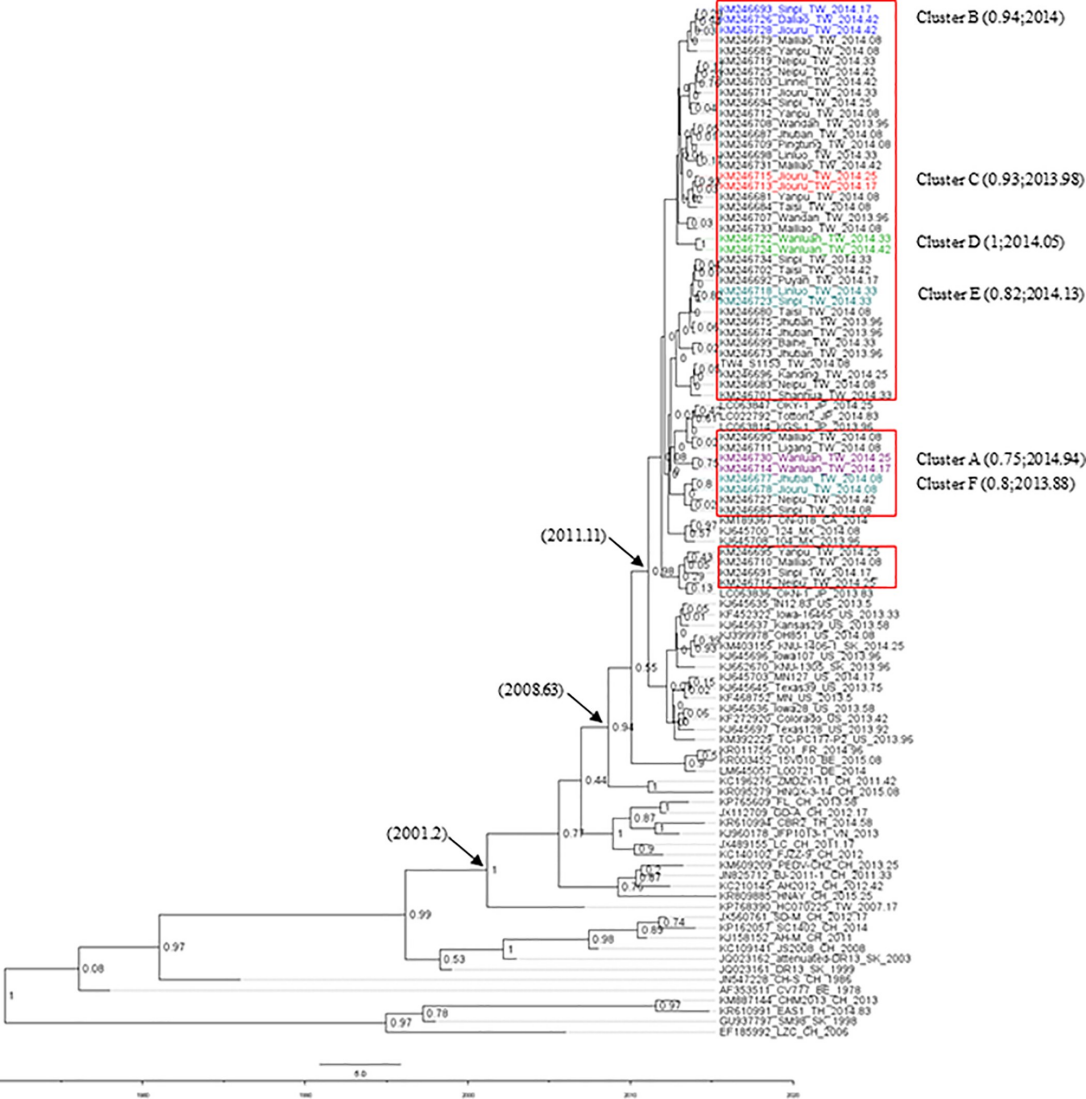
Data from PEDV99 pS-648 MCMC phylogenetic tree analysis using aligned sequences of 49 partial S genes collected in Taiwan, 49 whole genome sequences collected in other countries, and 1 Taiwan PEDV whole genome sequence. Maximum likelihood and Bayesian methods (MEGA 6.0 software) were used for tree construction. An interior branch test (1,000 replicates) was performed to determine internal node reliability. Scale bar indicates nucleotide substitutions per site. Monophyletic groups were identified as having bootstrap values >75%. Strains shown in box were collected in Taiwan.

The maximum-likelihood phylogenetic tree (Fig 1)was constructed from 49 global PEDV whole genome sequences. Sequences found in Taiwan clustered with 100% bootstraps containing viruses from other Asian countries and the US between 2011 and 2015. A correlation was determined between this cluster and the Genogroup 2 originally identified in the US. Among the Taiwan sequences, some of the viruses collected and identified in 2014 were strongly correlated with one another (bootstrap values >75%), while others were mixed with viral strains collected in other countries at different times, suggesting multiple transmission events (Fig 2 & Table 1).

**Table 1:** Results from MEGA6 DNA-DNA homology cluster analyses.

Cluster	Estimated time of Origin	Bootstrap Value (%)	Identity (nt/nt) (%)*	Accession Number	Geographic Origin
A	2014.94	0.75	645/648 (99%)	KM246714	Wanluan
				KM246730	Wanluan
B	2014	0.94	647/648 (99%)	KM246693	Sinpi
				KM246726	Daliao
				KM246728	Jiouru
C	2013.98	0.93	646/648 (99%)	KM246713	Jiouru
				KM246715	Jiouru
D	2014.05	1	645/648 (99%)	KM246722	Wanluan
				KM246724	Wanluan
E	2014.13	0.82	647/648 (99%)	KM246718	Linluo
				KM246723	Sinpi
F	2013.88	0.8	646/648 (99%)	KM246677	Jhutian
				KM246678	Jiouru

* BLAST calculations.

### Phylodynamic data for PEDV samples from different farms

**Fig 3:**
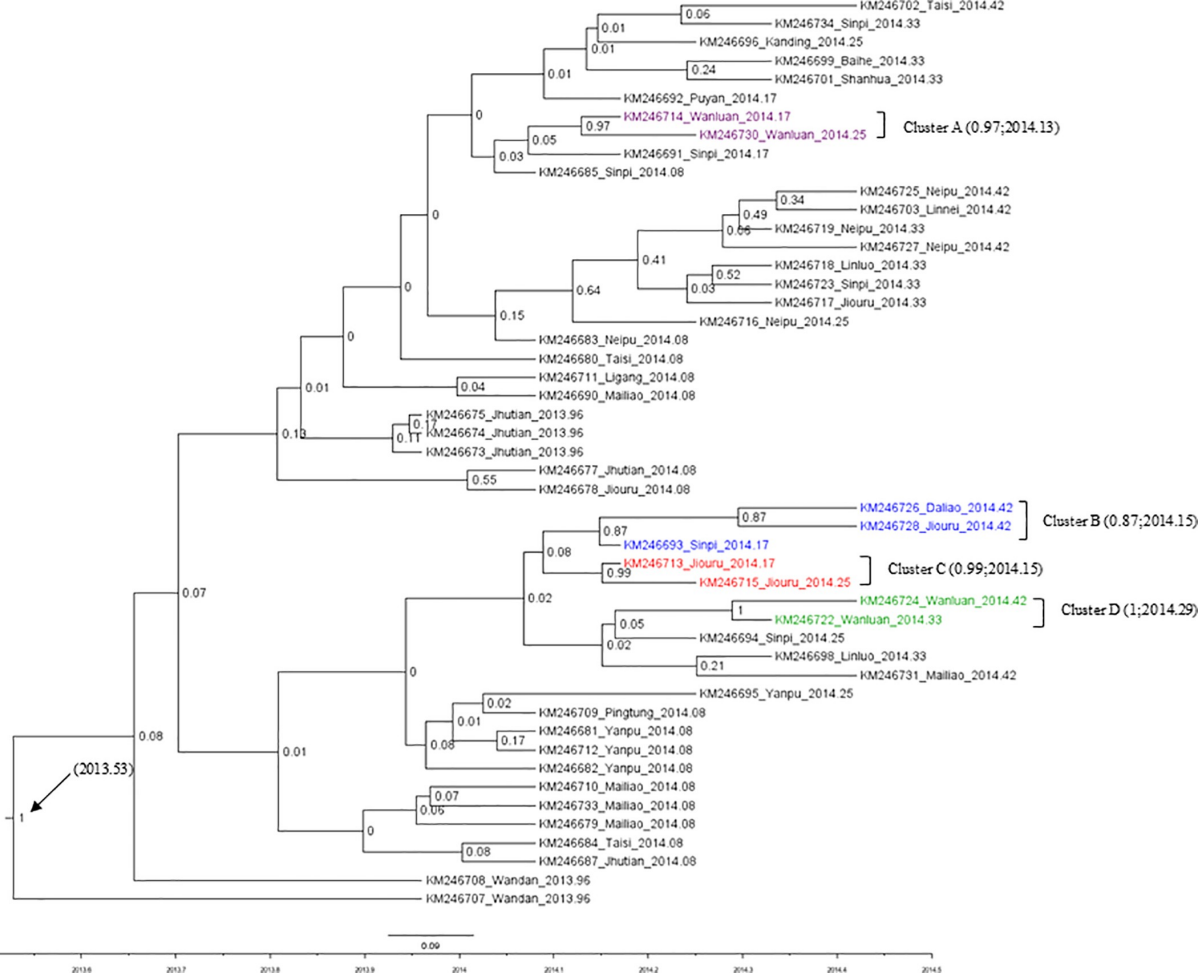
Times of origin of Taiwan PEDV viruses, identified by coalescent data analysis using maximum clade credibility trees [7] based on 49 partial S genes (positions 1468–2115) from PEDV samples collected in Taiwan. MCMC trees were generated by Bayesian Markov Chain Monte Carlo sampling using BEAST v1.8.2. Maximum clade credibility trees with temporal and spatial annotations were summarized with 10% burn-in removed using TreeAnnotator v1.8.2 in the BEAST package. Presentation figures were generated using FigTree v1.4.2.

To further evaluate transmission periods and to verify viral strain origins, all 49 partial S gene sequences determined from the Taiwan PEDV samples were used for phylodynamic data analyses (Fig 3). Four clusters (A, B, C and D) exhibited statistically significant posterior probabilities (>0.8), including 9 viral strains on 4 monophyletic branches. The most recent common ancestor (MRCA) for the 9 viral strains was traced to July 2013. All other coalescent tree sequences were determined as independent. Viral branches had MRCAs in 2013 or the spring of 2014, with none identified as statistically significant. Thus, the phylodynamic coalescent tree established for this study indicates the involvement of multiple virus strains in the 2014 PEDV outbreak.

### Phylogeographic tree representations of potential transmission routes

**Fig 4:**
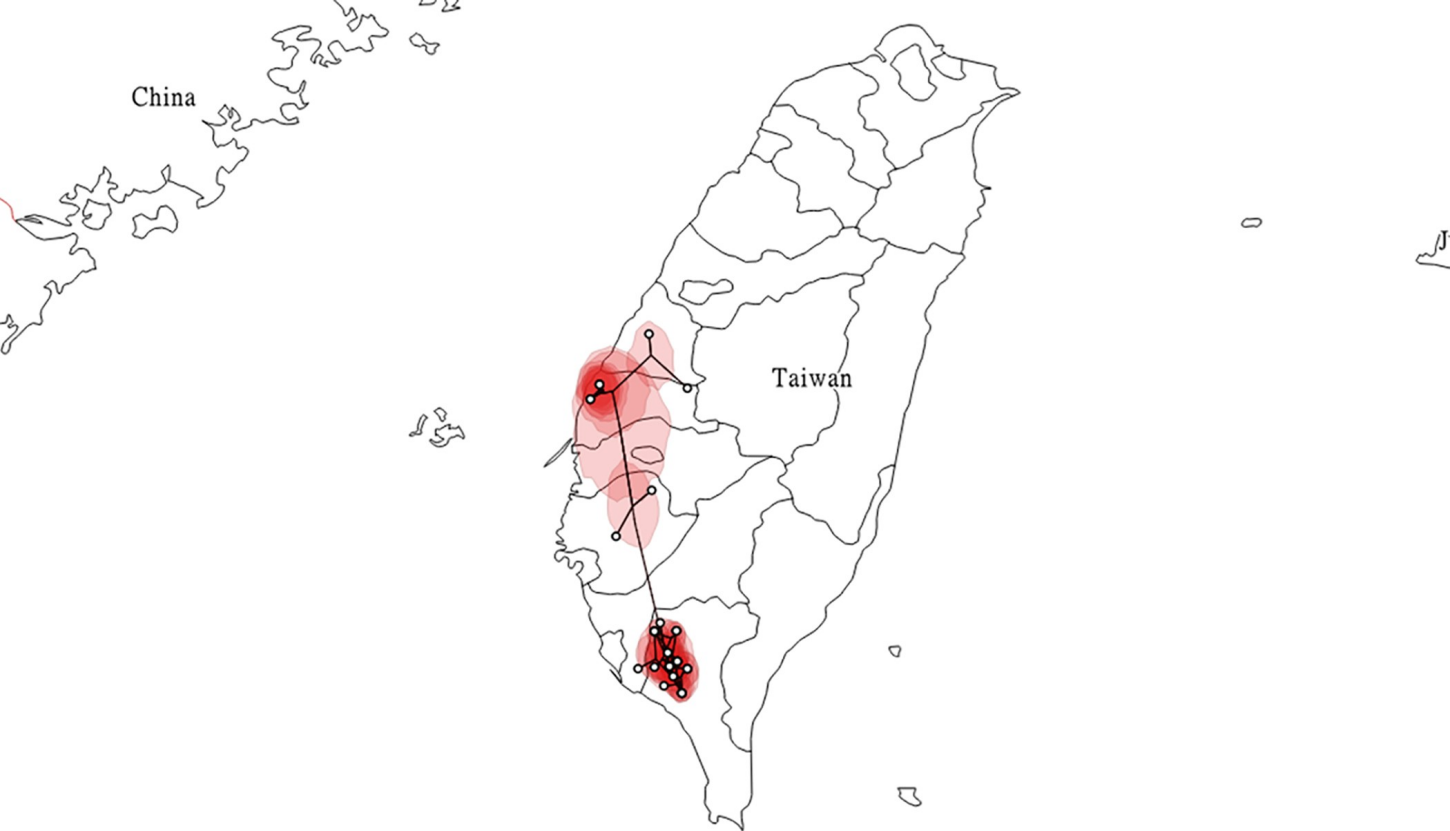
Phylogeographic map for 49 PEDV samples collected in Taiwan. Map was produced from partial S gene sequences identified from the 2013–2014 outbreak, constructed using SPREAD3 [32], and visualized using QGIS (v3.2.3). Cycle symbols indicate PEDV-positive case distribution as shown in S2 Table.

Results from our phylogeographic analysis were matched to a map of the country to specify the geographic boundaries of the Taiwan outbreak (Fig 4). Next, molecular sequence data were combined with isolation time data and geographic coordinates to determine the spatiotemporal distribution of Taiwan PEDV strains. Lineages were identified in several agricultural communities in the far south, with additional virus strains found in central Taiwan at approximately the same time. The southern infections were spatially closer to each other. The data indicate an absence of natural and artificial barriers restricting the spread of the virus.

### Risk map showing relationships among various factors

**Fig 5:**
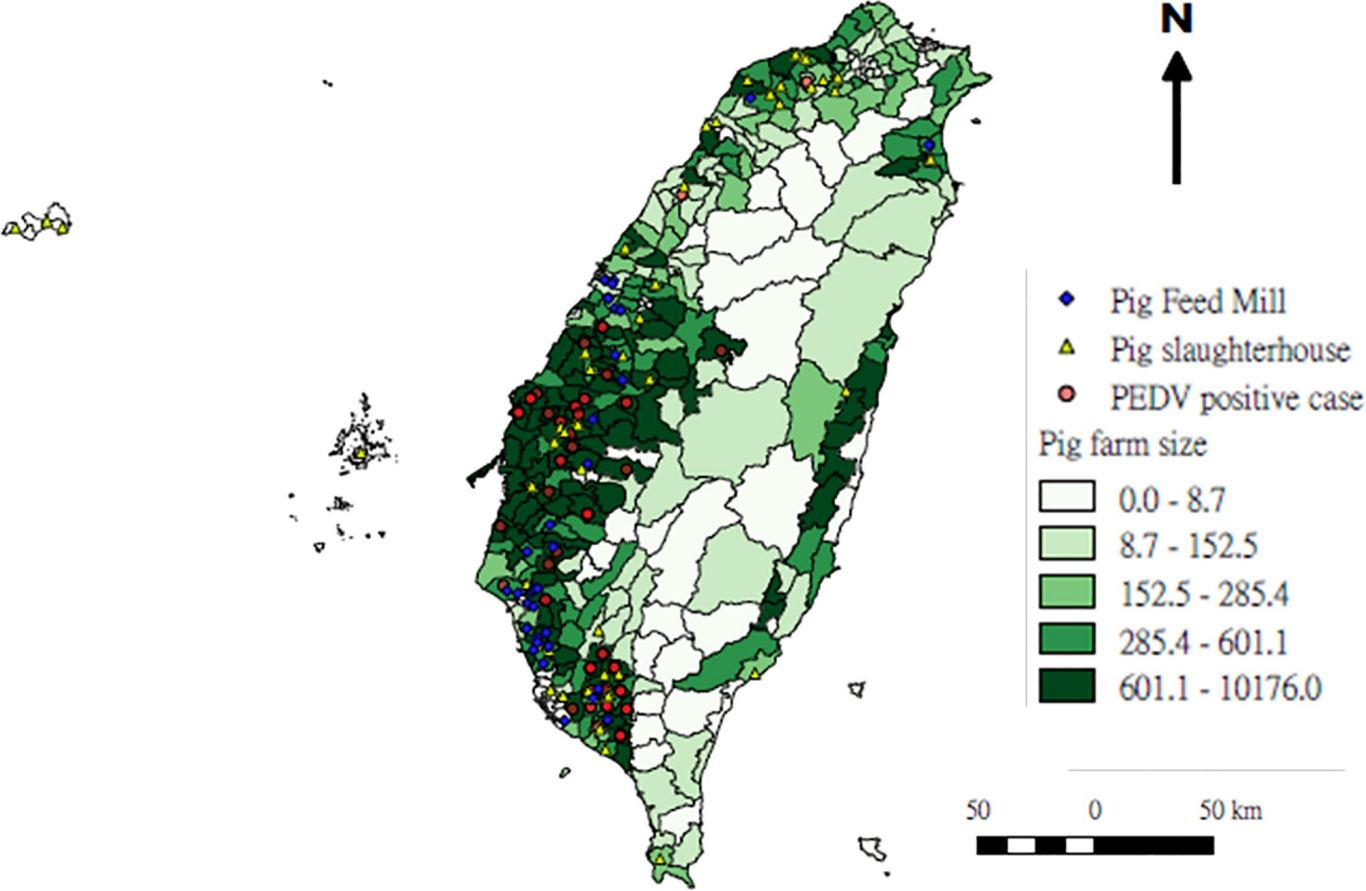
PEDV risk factor analysis data produced by geographic information system. Correlations between risk factors and PEDV-positive cases were determined using a mixed linear regression analysis and drawn using QGIS software.

The pig feed industry risk map shown was created to assist in the identification of significant risk factors associated with infections (Fig 5). Results from our GIS system analysis of positive cases indicate correlations between PEDV infection transmission and both pig farm size (number of pigs, not physical size) (0.000232, 95% CI [0.000102, 0.000362]) and slaughterhouse distribution (0.8043, 95% CI [0.4351, 1.1735]) (Table 2). Our data also indicate higher infection rates in counties with fewer pig feed mills (-0.7857, 95% CI [-1.2298, -0.3415]).

**Table 2:** Correlation data for numbers of PEDV cases and pig farm size, number of feed mills, and number of pig slaughterhouses.

	Correlation Coefficient	p-value	95% CI
Intercept	0.1114	0.1746	(-0.05021,0.2729)
Farm Size	0.000232	0.0005	(0.000102, 0.000362)
Slaughterhouse Number	0.8043	< .0001	(0.4351,1.1735)
Feed Mill Number	-0.7857	0.0006	(-1.2298, -0.3415)

## Discussion

Phylogeographic inferences are a potential tool for identifying the transmission and dissemination routes of PEDV and other potentially much deadlier infectious diseases. However, to date very few research efforts in Asia have utilized full genome sequencing for determining geographic structures due to the high costs and enormous amounts of computational time required for analyses [33, 34]. Several researchers have suggested using partial S genes for phylogenetic tree construction and for phylodynamic analyses specifically aimed at studying the genetic relatedness of PEDV strains [3, 10, 19]. In this study, we investigated temporal and geographic relationships among PEDV strains identified as having been transmitted among farms in Taiwan in 2014, using partial sequences from S genes extracted from porcine fetus samples and GenBank sequences as reference panels.

According to the phylogenetic tree we constructed based on these partial S gene sequences, the primary PEDV strain in Taiwan is related to the Genogroup 2 strain identified in samples collected in the US in 2013 [35]. As previously suggested, the most recently identified Taiwan PEDV strains have greater similarity with US strains than with Chinese or earlier Taiwanese strains [3, 36]. Transmission may have occurred as early as December 2013 [36], but the cross-border route to Taiwan remains unknown. Results from our phylogenetic analysis of PEDV viruses associated with the Taiwan outbreak confirm independence in multiple counties. Although these viruses share a common ancestry with the US Genogroup 2 PEDV, our coalescent tree data indicate that only 9 of the Taiwanese viruses were significantly clustered (Fig 3). Further, no recombination involving Taiwanese strains was observed in the present study.

Five independent PEDV strains were identified in Taiwan on or before September 16, 2013 in Wanda (KM246707 and KM246708) and Jhutian (KM246673, KM246674 and KM246675) townships. These are considered starting points for PEDV complex dissemination throughout sections of Taiwan up to May 15, 2014. According to a mix of phylogenetic and coalescent data (Table 2), clustering was limited to 3 counties in southern Taiwan (Wanluan, Jiouru and Daliao) (Fig 3), with most of the identified viruses existing independently. Combined, data for PEDV divergence during the Taiwan outbreak suggest a common ancestry shared by multiple virus lineages. Since that time period, no evidence has been found indicating outbreaks involving multiple virus strains in Taiwan. According to our phylogenetic analysis, most of the identified PEDV strains from the 2014 outbreak were independent, despite sharing a common ancestor. Multiple PEDV invasions from abroad were also identified in Japan during the 2014 outbreak [15, 37]. The widespread dissemination that followed presumably resulted from the movement of pigs, agricultural vehicles, farmers, farm visitors, commercial feed products, and other materials [15, 37].

Reproducing novel approaches used in the molecular and spatial surveillance of the porcine reproductive and respiratory syndrome virus (PRRSV) in the US and in PEDV studies in Japan [26, 38], we utilized a combination of phylogeographic and GIS approaches in our effort to profile the Taiwan PEDV outbreak. GIS has been used to investigate correlations between diseases and factors that include pig farm size, number of feed mills, and number of pig slaughterhouses in a specified geographic zone (Fig 5). We used a mixed linear regression to identify factors associated with the number of PEDV cases in Taiwan. Results indicate positive correlations between the number of cases and both slaughterhouse number and pig farm size, and a negative correlation with number of feed mills (Table 1). The highest concentration of PEDV cases was identified in a multi-county section of southern Taiwan characterized by a large number of pig farms with high animal densities. Our results are in agreement with findings from previous studies suggesting that excessive farm capacity (measured as the total number of pigs on a farm) is a risk factor for the spread of PEDV [5]. Further, aerosol transmission is considered a viable dissemination route in environments marked by high pig densities and close animal proximities [4]. Regarding the negative correlation between PEDV cases and number of feed mills, our data indicate that pig farms at the end of feed routes likely have higher probabilities of infections. Some reports suggest that vehicles used for the dual purposes of transporting swine to slaughterhouses and delivering feed to farms may increase the potential for PEDV due to feedbag contamination [5, 25, 39, 40]. Our finding of a high correlation between the number of PEDV cases and the number of pig slaughterhouses suggests that transport trucks may have been a factor in the 2014 Taiwan outbreak.

Other risk factors requiring further research include spray-dried porcine plasma (SDPP, an important blood-based component of nursery pig diets) and improper disposal procedures when pig corpses are collected and sent to rendering plants. There is a clear need to collect more data on feed truck routes, SDPP supplement distribution, rendering plant procedures (especially delivery), and slaughterhouse processes when trying to identify the sources of various strains in Taiwan. Due to the potential for significant financial losses, there is a strong need to act on these and other possible factors before detailed studies can be designed, funded, and completed. Farms, slaughterhouses, and feed suppliers in counties with high pig densities need to immediately enhance their biosecurity measures to prevent future PEDV outbreaks, and greater effort is required to monitor potential transmission routes.

## Supporting information

S1 Table List of 49 PEDV whole genome sequences. (DOCX)

S2 Table List of 49 Taiwan PEDV partial S (648 nt of PEDV S1 gene position 1468–2115). (DOCX)
