# Supplementary material for: Phylogeographic investigation of 2014 porcine epidemic diarrhea virus (PEDV) transmission in Taiwan
Source: PLoS One. 2019 Mar 6;14(3):e0213153. doi: 10.1371/journal.pone.0213153 (PMC6402684; doi:10.1371/journal.pone.0213153)
Supplement: S1 Table — (DOCX) [file pone.0213153.s001.docx]

**S1 Table: List of 49 PEDV whole genome sequences**

| Accession no. | Strain | Year | Country | Genogroup ([Huang et al., 2013](#_ENREF_12)) | S INDEL | North American clade ([Vlasova et al., 2014](#_ENREF_27)) |
| --- | --- | --- | --- | --- | --- | --- |
| AF353511 | CV777 | 1978 | Belgium (BE) | 1a | S INDEL (Classical) | - |
| EF185992 | LZC | 2006 | China (CH) | 1a | S INDEL (Classical) | - |
| GU937797 | SM98 | 1998 | South Korea | 1a | S INDEL (Classical) | - |
| JN547228 | CH/S | 1986 | China (CH) | R | S INDEL (Classical) | - |
| JN825712 | BJ-2011-1 | 2011 | China (CH) | 2a | - | - |
| JQ023161 | virulent DR13 | 1999 | South Korea (SK) | R | S INDEL (Classical) | - |
| JQ023162 | attenuated DR13 | 2003 | South Korea (SK) | 1b | S INDEL (Classical) | - |
| JX112709 | GD-A | 2012 | China (CH) | 2b | - | - |
| JX489155 | LC | 2011 | China (CH) | 2b | - | - |
| JX560761 | SD-M | 2012 | China (CH) | 1b | S INDEL (Classical) | - |
| KC109141 | JS2008 | 2008 | China (CH) | 1b | S INDEL (Classical) | - |
| KC140102 | CH/FJZZ-9/2012 | 2012 | China (CH) | 2a | - | - |
| KC196276 | CH/ZMDZY/11 | 2011 | China (CH) | 2a | - | - |
| KC210145 | AH2012 | 2012 | China (CH) | 2a | - | - |
| KF272920 | USA/Colorado/2013 | 2013 | USA (US) | 2a | - | North American clade II |
| KF452322 | USA/Iowa/16465/2013 | 2013 | USA (US) | 2a | - | North American clade I |
| KF468752 | MN | 2013 | USA (US) | 2a | - | North American clade I |
| KJ158152 | AH-M | 2011 | China (CH) | 1b | S INDEL (Classical) | - |
| KJ399978 | OH851 | 2014 | USA (US) | 2a | - | North American clade II |
| KJ645635 | USA/Indiana12.83/2013 | 2013 | USA (US) | 2a | - | North American clade II |
| KJ645636 | USA/Iowa28/2013 | 2013 | USA (US) | 2a | - | North American clade I |
| KJ645637 | USA/Kansas29/2013 | 2013 | USA (US) | 2a | - | North American clade II |
| KJ645645 | USA/Texas39/2013 | 2013 | USA (US) | 2a | - | North American clade I |
| KJ645696 | USA/Iowa107/2013 | 2013 | USA (US) | 2a | - | North American clade II |
| KJ645697 | USA/Texas128/2013 | 2013 | USA (US) | 2a | - | North American clade II |
| KJ645700 | MEX/124/2014 | 2014 | Mexico (MX) | 2a | - | North American clade I |
| KJ645703 | USA/Minnesota127/2014 | 2014 | USA (US) | 2a | - | North American clade I |
| KJ645708 | MEX/104/2013 | 2013 | Mexico (MX) | 2a | - | North American clade II |
| KJ662670 | KNU-1305 | 2013 | South Korea (SK) | 2a | - | North American clade I |
| KJ960178 | VN/JFP1013_1/2013 | 2013 | Vietnam (VN) | 2b | - | - |
| KM189367 | ON-018 | 2014 | Canada (CA) | 2a | - | North American clade I |
| KM392229 | TC PC177-P2 | 2013 | USA (US) | 2a | - | North American clade I |
| KM403155 | KNU-1406-1 | 2014 | South Korea (SK) | 2a | S INDEL (New) | North American clade II |
| KM609209 | PEDV-CHZ | 2013 | China (CH) | 2a | - | - |
| KM887144 | CHM2013 | 2013 | China (CH) | 1a | S INDEL (Classical) | - |
| KP162057 | SC1402 | 2014 | China (CH) | 1b | S INDEL (Classical) | - |
| KP765609 | FL2013 | 2013 | China (CH) | 2b | - | - |
| KR003452 | 15V010/BEL/2015 | 2015 | Belgium (BE) | 2a | S INDEL (New) | North American clade II |
| KR011756 | FR/001/2014 | 2014 | France ([Lowe et al.](#_ENREF_18)) | 2a | S INDEL (New) | North American clade II |
| KR095279 | CH/HNQX-3/14 | 2015 | China (CH) | 2a | - | - |
| KR610991 | EAS1 | 2014 | Thailand (TH) | 1a | S INDEL (Classical) | - |
| KR610994 | CBR2 | 2014 | Thailand (TH) | 2b | - | - |
| KR809885 | CH/HNAY/2015 | 2015 | China (CH) | 2a | - | - |
| LC022792 | Tottori2/JPN/2014 | 2014 | Japan (JP) | 2a | - | North American clade I |
| LC063814 | KGS-1/JPN/2013 | 2013 | Japan (JP) | 2a | - | North American clade I |
| LC063836 | OKN-1/JPN/2013 | 2013 | Japan (JP) | 2a | - | North American clade II |
| LC063847 | OKY-1/JPN/2014 | 2014 | Japan (JP) | 2a | S INDEL (New) | North American clade II |
| LM645057 | L00721/GER/2014 | 2014 | Germany (DE) | 2a | S INDEL (New) | North American clade II |
| KJ434297 | TW4 | 2014 | Taiwan (TW) | 2a | - | North American clade I |
