# Supplementary material for: Phylogeographic investigation of 2014 porcine epidemic diarrhea virus (PEDV) transmission in Taiwan
Source: PLoS One. 2019 Mar 6;14(3):e0213153. doi: 10.1371/journal.pone.0213153 (PMC6402684; doi:10.1371/journal.pone.0213153)
Supplement: S2 Table — (DOCX) [file pone.0213153.s002.docx]

**S2 Table: List of 49 Taiwan PEDV partial S sequences (648 nt of PEDV S1 gene position 1468-2115)**

| *Access* number | Strain | Collection date | Region |
| --- | --- | --- | --- |
| KM246673 | PEDV-102-565/2013/Ak | 12/27/2013 | Pingtung_Jhutian |
| KM246674 | PEDV-102-566/2013/Ak | 12/27/2013 | Pingtung_Jhutian |
| KM246675 | PEDV-102-567/2013/Ak | 12/27/2013 | Pingtung_Jhutian |
| KM246677 | PEDV-103-009/2014/Z | 1/6/2014 | Pingtung_Jhutian |
| KM246678 | PEDV-103-010/2014/Aa | 1/6/2014 | Pingtung_Jiouru |
| KM246679 | PEDV-103-014-10/2014/C | 1/13/2014 | Yunlin_Mailiao |
| KM246680 | PEDV-103-015-2/2014/C | 1/13/2014 | Yunlin_Taisi |
| KM246681 | PEDV-103-016/2014/N | 1/13/2014 | Pingtung_Yanpu |
| KM246682 | PEDV-103-020/2014/N | 1/13/2014 | Pingtung_Yanpu |
| KM246683 | PEDV-103-024/2014/E | 1/14/2014 | Pingtung_Neipu |
| KM246684 | PEDV-103-032-2/2014/O | 1/15/2014 | Yunlin_Taisi |
| KM246685 | PEDV-103-036-3/2014/P | 1/15/2014 | Pingtung_Sinpi |
| KM246687 | PEDV-103-044-3/2014/Am | 1/20/2014 | Pingtung_Jhutian |
| KM246690 | PEDV-103-116/2014/C | 1/27/2014 | Yunlin_Mailiao |
| KM246691 | PEDV-103-120/2014/G | 2/10/2014 | Pingtung_Sinpi |
| KM246692 | PEDV-103-127/2014/Ac | 2/13/2014 | Changhua_Puyan |
| KM246693 | PEDV-103-132-1/2014/P | 2/13/2014 | Pingtung_Sinpi |
| KM246694 | PEDV-103-159/2014/P | 3/10/2014 | Pingtung_Sinpi |
| KM246695 | PEDV-103-174/2014/Ad | 3/13/2014 | Pingtung_Yanpu |
| KM246696 | PEDV-103-181/2014/Ae | 3/18/2014 | Pingtung_Kanding |
| KM246698 | PEDV-103-232/2014/Af | 4/1/2014 | Pingtung_Linluo |
| KM246699 | PEDV-103-236/2014/An | 4/2/2014 | Tainan_Baihe |
| KM246701 | PEDV-103-295/2014/J | 4/23/2014 | Tainan_Shanhua |
| KM246702 | PEDV-103-314/2014/U | 5/2/2014 | Yunlin_Taisi |
| KM246703 | PEDV-103-329/2014/Ah | 5/7/2014 | Yunlin_Linnei |
| KM246707 | PEDV-S-1140/2013/W | 12/23/2013 | Pingtung_Wandan |
| KM246708 | PEDV-S-1141/2013/W | 12/23/2013 | Pingtung_Wandan |
| KM246709 | PEDV-S-1171/2014/F | 1/13/2014 | Pingtung_Pingtung |
| KM246710 | PEDV-S-1187/2014/Am | 1/21/2014 | Yunlin_Mailiao |
| KM246711 | PEDV-S-1197/2014/H | 1/27/2014 | Pingtung_Ligang |
| KM246712 | PEDV-S-1199/2014/Ar | 1/27/2014 | Pingtung_Yanpu |
| KM246713 | PEDV-S-1228/2014/A | 2/12/2014 | Pingtung_Jiouru |
| KM246714 | PEDV-S-1235/2014/D | 2/18/2014 | Pingtung_Wanluan |
| KM246715 | PEDV-S-1253/2014/A | 3/4/2014 | Pingtung_Jiouru |
| KM246716 | PEDV-S-1256/2014/Q | 3/5/2014 | Pingtung_Neipu |
| KM246717 | PEDV-S-1294-2/2014/A | 4/2/2014 | Pingtung_Jiouru |
| KM246718 | PEDV-S-1304/2014/Ag | 4/18/2014 | Pingtung_Linluo |
| KM246719 | PEDV-S-1306/2014/B | 4/21/2014 | Pingtung_Neipu |
| KM246722 | PEDV-S-1319/2014/K | 4/28/2014 | Pingtung_Wanluan |
| KM246723 | PEDV-S-1324/2014/I | 4/29/2014 | Pingtung_Sinpi |
| KM246724 | PEDV-S-1336/2014/K | 5/7/2014 | Pingtung_Wanluan |
| KM246725 | PEDV-S-1340/2014/B | 5/8/2014 | Pingtung_Neipu |
| KM246726 | PEDV-S-1350/2014/M | 5/25/2014 | Kaohsiung_Daliao |
| KM246727 | PEDV-S-1360/2014/Ap | 5/22/2014 | Pingtung_Neipu |
| KM246728 | PEDV-S-1375/2014/A | 5/29/2014 | Pingtung_Jiouru |
| KM246730 | PEDV-S-1240/2014/D | 3/3/2014 | Pingtung_Wanluan |
| KM246731 | PEDV-103-399-1/2014/V | 5/30/2014 | Yunlin_Mailiao |
| KM246733 | PEDV-103-109/2014/C | 1/20/2014 | Yunlin_Mailiao |
| KM246734 | PEDV-103-292-2/2014/P | 4/23/2014 | Pingtung_Sinpi |
